# Is impaired energy regulation the core of the metabolic syndrome in various ethnic groups of the USA and Taiwan?

**DOI:** 10.1186/1472-6823-10-11

**Published:** 2010-06-01

**Authors:** Mark L Wahlqvist, Hsing-Yi Chang, Chu-Chih Chen, Chih-Cheng Hsu, Wan-Chi Chang, Wuan-Szu Wang, Chao A Hsiung

**Affiliations:** 1 Division of Health Policy Research and Development, Institute of Population Health Sciences, National Health Research Institutes, No. 35, Keyan Road, Zhunan, Miaoli County 35053, Taiwan; 2 Division of Biostatistics and Bioinformatics, Institute of Population Health Sciences, National Health Research Institutes, No. 35, Keyan Road, Zhunan, Miaoli County 35053, Taiwan

## Abstract

**Background:**

The metabolic syndrome (MetS) concept is widely used in public health and clinical settings without an agreed pathophysiology. We have re-examined the MetS in terms of body fuels, so as to provide a coherent cross-cultural pathogenesis.

**Methods:**

National Health and Nutrition Examination Survey (NHANES 2001-2) with n = 2254 and Taiwanese National Health Interview Survey (NHIS) sub-set for hypertension, hyperglycemia and hyperlipidemia assessment (TwSHHH 2002), n = 5786, were used to compare different ethnicities according to NCEP-ATPIII (NCEP-tw) criteria for METS. Exploratory factor analysis (EFA) using principal components (PC) was employed to differentiate and unify MetS components across four ethnicities, gender, age-strata, and urban-rural settings.

**Results:**

The first two factors from the PC analysis (PCA) accounted for from 55.2% (non-Hispanic white) to 63.7% (Taiwanese) of the variance. Rotated factor loadings showed that the six MetS components provided three clusters: the impaired energy regulation (IER) components (waist circumference, WC, fasting triglycerides, TG, and fasting plasma glucose, FPG), systolic and diastolic blood pressures (BPs), and HDL-cholesterol, where the IER components accounted for 25-26% of total variance of MetS components. For the three US ethnic subgroups, factor 1 was mainly determined by IER and HDL-cholesterol, and factor 2 was related to the BP components. For Taiwanese, IER was determinant for both factors, and BPs and HDL-cholesterol were related to factors 1 and 2 respectively.

**Conclusions:**

There is a MetS core which unifies populations. It comprises WC, TG and FPG as a core, IER, which may be expressed and modulated in various second order ways.

## Background

The metabolic syndrome (MetS) was conceptualized on the basis that a cluster of metabolic phenomena could be observed in those prone to cardiovascular disease ahead of frank diabetes. It combined features of possible pathogenesis with risk evaluation. Its definition has been in evolution, but those currently used include that of the International Diabetes Federation (IDF) in 2005 [1,2] and the joint NHLB (National Heart Lung and Blood) Institute/AHA (American Heart Association) definition of 2004 [3], based on the NCEP-ATP III (National Cholesterol Education Program-Adult Treatment Panel III) [4]. The latter has also been modified in Taiwan as NCEP-tw [5]. Both clinical and public health utility have been in mind. However, a 'syndrome' is usually the way in which clinicians articulate what they observe in patients as a set of symptoms and signs, but where etiology or pathogenesis is uncertain. The field of enquiry into the MetS has grown rapidly without settling the matter of the syndrome being a coherent disorder or disease for clinicians or, for public health workers, an entity with 'inputs' and scope for prevention and with 'outputs' in monitoring and surveillance. Despite these limitations, Japanese health authorities have recently embarked on a national program to encourage citizens to have their MetS assessed and to engage in rigorous efforts to 'normalize' it. Unfortunately, definitions differ in different places by 'cut-off' points rather than continua; the basis of these 'cut-offs' is rarely on the basis of all-cause morbidity or mortality; and the relationship of measurements like waist circumference to underlying anatomy not well-characterized. Interestingly, in Japan, imaging techniques have allowed waist circumference to be related to intra-abdominal fat so that the usual gender acceptance of a greater girth for men than women is reversed [6].

There have been efforts to create a unified explanatory model for this syndrome. The best known of these is that the MetS is a disorder of insulin sensitivity i.e. basically 'insulin resistance' and its sequelae, however it may be caused, as suggested by the IDF [1].

It could be argued that the anatomico-physiological basis of the syndrome is in the splanchnic region [7] with disordered energy metabolism [8]. This region has a portal circulation which links the venous drainage of the gut, omental fat, pancreas and spleen to the liver which, in turn, drains into the systemic venous system (the inferior vena cava). It constitutes the metabolic focus for regulation of energy balance, albeit with neuro-endocrine control, and accounts for some of the MetS variables while other variables are a consequence of this disorder [9]. Omental fat (which contributes to abdominal or waist circumference) represents a very metabolically active site for fat storage with a free fatty acid (FFA) flux which acts a regulator of hepatic gluconeogenesis [10] and of hepatic triglyceride (TG) and its very low density lipoprotein (VLDL) TG synthesis [11]. The major function of VLDL TG is to transport energy to peripheral tissues [12]. FFA play a role at the periphery as well in regulating glucose uptake and may be as potent as insulin, but in the reverse direction [13]. Thus, waist circumference as a surrogate for omental fat and FFA flux, serum triglycerides and fasting glucose (representing nocturnal hepatic gluconeogenesis) could serve as a composite index of energy metabolism or its disorders. There is, nevertheless, debate about whether waist circumference adds any additional value to the assessment of body fatness by use of BMI (body mass index) [14], yet the distribution of body fat is relevant to the pathogenesis of various clinical disorders, notably diabetes and cardiovascular disease and, in turn, to mortality although under review by some authors [15]. In the evaluation of the pathology of fat distribution, it is a consideration as to what extent WC represents visceral (elemental) fat [16], which is the metabolically relevant fat in the splanchnic region.

The MetS and energy dysmetabolism are modulated by a number of factors including the psycho-social [9,17] and environmental; locality [18]; food systems; physical activity patterns, biological rhythms (sleep, leisure and work, and sunlight exposure which affects vitamin D status) and pollutants [12]. Their relevant consequences include inflammatory [19-21], and vascular pathways [21].

There are often striking differences in the presentations of the MetS in Western and Eastern populations. We have taken advantage of such differences between the USA and Taiwan to test for hypothesis generation. In the case of the USA, we have used the National Health and Examination Survey (NHANES) of 2001-2002 data [22] and in the case of Taiwan, we have used a sub-set of the Taiwanese Health Survey, the "Taiwan Three High Prevalence Survey on Hypertension, Hyperglycemia, and Hyperlipidemia" (TwSHHH) of 2002 [23].

Exploratory factor analysis (EFA) has been used for hypothesis generation. It is a multivariate correlation method to reduce a number of inter-correlated variables into smaller sets of factors with less or no association; it has been applied to study the clustering of metabolic abnormalities that contribute to the metabolic syndrome [18,20,24,25]. Here we have used EFA to differentiate major factors derived from the six components of the MetS as continuous variables. A single factor structure or multi-factorial structure can suggest a common or heterogeneous pathogenetic background, respectively, as underlying the clinical manifestations of the syndrome [25]. We sought invariance in clustering patterns of the estimated factor loadings (or communalities) across the four ethnic subgroups. Specifically, we tested the proposition that there is a core to the metabolic syndrome which reflects energy dysregulation. This would manifest as at least one factor which could describe energy storage and transport, possibly by way of abdominal fatness for storage and as a dynamic source of FFA for energy transport along with both fasting glucose and triglycerides as substrates too.

## Methods

### Subjects and measurements

The study was approved by the Institutional Review Board (ethics committee) of the NHRI (National Health Research Institutes) of Taiwan.

Two datasets, one from each of Taiwan and the US were used for this study. The Taiwan data came from the Taiwanese Survey on Hypertension, Hyperglycemia, and Hyperlipidemia (TwSHHH) in year 2002 [23], a subset of the national health interview survey (NHIS) conducted in 2001 [26]. Community-based subjects, aged 17-91 years were identified through the national household registry. There were no exclusions on health grounds, but residential institutions were not included in the sampling frame as the study was community based. However, those with missing or biologically implausible data were not included in the analysis. NHIS incorporated a multistage stratified systematic sampling scheme. It first divided 359 townships/districts of Taiwan into seven strata according to their geographical location and degree of urbanization or rural location. Townships or districts in each stratum were selected with a selection probability proportional to their size (PPS). In each selected township/district, *lin*s (the smallest administrative unit) were selected with PPS. Four households were selected randomly from each selected *lin*, with each member of these households to be interviewed. The response rate for households was 91.1%, and it was 94.2% for individuals [24]. The design resulted in an equal probability sample. No sampling weights were required in the estimation. Anthropometric measurements were taken. Subjects were asked to avoid walking, running or lifting heavy things 30 minutes before blood pressures were measured. After they sat down for measuring blood pressures, they were asked to rest for 5 to 10 minutes. Two blood pressure readings were made with a calibrated mercury sphygmomanometer in the sitting position using a binaural stethoscope and simultaneous readings by 2 observers who recorded the findings independently. Readings were accepted only if they did not differ by more than 2 mmHg If the 2 measurements separated in time differed by more than 10 mmHg, a third reading was obtained. The average of the closest 2 readings was used. Subjects were instructed to fast for 8 hours or more before the blood samples were taken. NaF plasma was collected for fasting glucose analysis, and serum was collected for measurements of lipids. Blood samples were transported at -10°C in dry ice to the central laboratory, stored at -20°C and analyzed within 2 weeks. Fasting plasma glucose (FPG) was analyzed by the glucose oxidase method using an automated system (Vitros 550/750, Ortho-Clinical Diagnostic Inc., Johnson and Johnson Company, Rochester, NY, USA).

The other dataset was the NHANES which comes from interviews, examinations, and laboratory tests based on blood and urine samples http://www.cdc.gov/nchs/nhanes.htm[22]. The 2001-2002 data were used for this study. Subjects were 18-85 years old, community-based, irrespective of health status, similar to the Taiwanese sample. Anthropometric, blood pressure recording, blood sampling and analyte measurements were subject to similar standardisation as used in Taiwan. In the case of BP, three and sometimes 4 BP determinations (systolic and diastolic) were taken in a mobile examination center (MEC) and during home examinations on all eligible individuals using a mercury sphygmomanometer; two physicians (MEC setting) and 2 health technologists (Home Examination setting) were trained to collect NHANES BP data [22].

As with the Taiwanese sample, when data were missing, or biologically implausible, the subject was not included in the analyses.

When examining the relationships between measurements, sampling weights were not necessary

### Statistical Analysis

Descriptive statistics, such as mean and standard deviation, were used to present the characteristics of the study populations. An EFA using the method of principal components [27] was applied to assess the factor structure of the six components: WC, FPG, TG, HDL, SBP, and DBP of the metabolic syndrome for all four ethnic groups: Taiwanese, non-Hispanic white, non-Hispanic black, and Mexican-American. Among the first two factors from principal component analysis (PCA), relative proportions of the total variance of the six standardized MetS components were estimated using the procedure PROC FACTOR from SAS software (SAS version 9.1. 2002; SAS Institute, Cary NC, USA). The factor loadings were then rotated using the *varimax *criterion for the clustering patterns of the six components from the first two factors for each of the ethnic groups. To assess the sensitivity of the estimated proportions, for each of the four ethnic groups, the EFA procedure was applied repeatedly for their communalities in each of the one thousand resampled datasets by bootstrapping [28]. The 95% confidence interval (CI) of the clustered components was then obtained by taking the 25^th ^and 975^th ^millile of the sorted added-up communalities of the corresponding clustered components among one thousand bootstrapped datasets.

## Results

### Descriptive statistics

The average ages and the metabolic components for the several ethnic groups are presented in Table 1. The average age of the non-Hispanic whites was 50.3 ± 20 years, whereas those of other ethnic groups was around 40 years old. Taiwanese had lower values for most of the components, except diastolic blood pressure and HDL (which were higher). That resulted in lower prevalence of abnormality for each component as well as for the metabolic syndrome than in other ethnic groups (Table 2). It is also noteworthy that Taiwanese subjects were the shortest and had the lowest Body Mass Indices (BMIs).

**Table 1:** Comparisons of the MetS components of Taiwanese (urban and rural) and the different US ethnic groups

Variables	Ethnic Groups						*P*-value^†^
		
	Taiwanese			US non-Hispanic whites	US non-Hispanic Black	US Mexican Americans	
					
	Urban	Rural	All				
**All**	**N = 3650**	**N = 2136**	**N = 5786**	**N = 1248**	**N = 457**	**N = 549**	

Age (years)	42.3 ± 15.4	42.9 ± 16.4	42.5 ± 15.7	50.3 ± 20.0	40.8 ± 18.0	40.5 ± 18.8	< .0001
WC (cm)	79.4 ± 11.3	79.9 ± 11.2	79.6 ± 11.3	96.7 ± 15.2	94.3 ± 17.3	95.4 ± 14.2	< .0001
Glucose (mg/dl)	92.5 ± 25.6	95.5 ± 29.1 **	93.6 ± 27.0	102.4 ± 29.0	101.7 ± 45.1	106.7 ± 37.9	< .0001
Triglycerides(mg/dl)	123.0 ± 79.4	132.1 ± 91.1 **	126.3 ± 84.0	157.4 ± 178.8	106.1 ± 69.8	156.3 ± 118.4	< .0001
HDL (mg/dl)	56.1 ± 14.8	54.5 ± 14.8 **	55.5 ± 14.8	52.5 ± 16.2	53.7 ± 16.3	49.4 ± 13.8	< .0001
LDL (mg/dl)	114.7 ± 27.0	116.4 ± 26.9 **	115.3 ± 27.0	122.0 ± 35.6	117.6 ± 36.8	115.8 ± 33.1	< .0001
Total Cholesterol(mg/dl)	182.7 ± 36.8	183.5 ± 36.5	183.0 ± 36.7	204.1 ± 44.1	192.4 ± 43.0	195.1 ± 41.0	< .0001
SBP (mm Hg)	113.7 ± 17.4	116.4 ± 17.6 **	114.7 ± 17.5	124.3 ± 20.2	125.3 ± 20.6	120.2 ± 18.9	< .0001
DBP (mm Hg)	74.5 ± 11.2	75.3 ± 11.2 **	74.8 ± 11.2	71.1 ± 11.4	73.5 ± 13.5	68.8 ± 11.4	< .0001
BMI (kg/m^2^)	23.1 ± 3.5.	23.3 ± 3.7 **	23.1 ± 3.6.	27.7 ± 5.9	28.4 ± 7.0	28.0 ± 5.7	< .0001
Weight (Kg)	61.5 ± 11.5	61.7 ± 11.7	61.5 ± 11.6	79.6 ± 19.0	82.2 ± 20.2	75.1 ± 17.5	< .0001
Height (cm)	162.9 ± 8.1	162.3 ± 7.9 **	162.6 ± 8.0	169.4 ± 10.0	170.4 ± 9.8	163.7 ± 9.1	< .0001
HT drug (%)	7.5	8.1	7.7	12.3	5.9	6.0	< .0001
DM drug (%)	3.1	3.3	3.2	5.5	8.1	9.3	< .0001
Lipid drug (%)	1.7	1.6	1.7	14.9	7.7	8.9	< .0001
Current smoker ^¥^(%)	18.8	22.1 **	20.0	22.1	26.5	21.1	0.0129

**Males**	**N = 1746**	**N = 1081**	**N = 2827**	**N = 602**	**N = 226**	**N = 266**	

Age (years)	42.5 ± 16.0	44.2 ± 17.0 **	43.1 ± 16.4	50.5 ± 19.3	40.5 ± 17.4	40.8 ± 19.1	< .0001
WC (cm)	84.2 ± 10.7	84.4 ± 10.2	84.3 ± 10.5	100.6 ± 14.0	92.2 ± 16.6	96.6 ± 13.3	< .0001
Glucose (mg/dl)	93.5 ± 27.4	96.5 ± 30.0 **	94.6 ± 28.5	106.8 ± 33.9	102.8 ± 46.8	109.9 ± 35.0	< .0001
Triglycerides(mg/dl)	139.1 ± 88.5	151.4 ± 103.6 **	143.8 ± 94.7	172.2 ± 238.3	111.0 ± 78.9	162.4 ± 138.1	< .0001
HDL (mg/dl)	52.1 ± 14.4	50.6 ± 14.7 **	51.5 ± 14.6	45.5 ± 12.0	50.9 ± 14.7	44.0 ± 10.7	< .0001
LDL (mg/dl)	116.8 ± 26.7	117.2 ± 26.6	113.7 ± 27.1	122.4 ± 36.0	117.1 ± 36.3	119.2 ± 34.2	0.0010
Total Cholesterol (mg/dl)	182.6 ± 35.9	182.4 ± 36.4	183.4 ± 37.2	199.7 ± 46.3	190.2 ± 43.6	192.9 ± 40.1	< .0001
SBP (mm Hg)	117.5 ± 15.9	120.7 ± 16.7 **	118.9 ± 16.3	124.7 ± 17.0	127.3 ± 20.0	123.0 ± 16.5	< .0001
DBP (mm Hg)	77.7 ± 10.7	78.3 ± 11.1	77.9 ± 10.9	73.0 ± 11.0	76.0 ± 13.6	69.7 ± 12.1	< .0001
BMI (kg/m^2^)	23.7 ± 3.4	23.9 ± 3.4	23.7 ± 3.4	27.9 ± 5.4	26.6 ± 5.7	27.6 ± 4.9	< .0001
Weight (Kg)	67.7 ± 10.8	67.4 ± 10.7	67.6 ± 10.7	87.0 ± 17.8	84.2 ± 19.3	80.0 ± 16.6	< .0001
Height (cm)	168.9 ± 6.1	167.8 ± 6.0 **	168.4 ± 6.1	176.7 ± 7.4	177.8 ± 6.9	170.0 ± 7.2	< .0001
HT drug (%)	7.6	9.2	8.2	12.5	6.6	5.6	0.0011
DM drug (%)	3.6	3.6	3.6	6.1	7.5	9.0	< .0001
Lipid drug (%)	1.7	1.6	1.7	14.8	8.4	7.5	< .0001
Current smoker ^¥^(%)	36.1	41.4	38.2	24.6	31.6	27.1	< .0001

**Females**	**N = 1904**	**N = 1055**	**N = 2959**	**N = 646**	**N = 231**	**N = 283**	

Age (years)	42.1 ± 14.8	41.6 ± 15.6	41.9 ± 15.0	50.1 ± 20.6	41.1 ± 18.9	40.2 ± 18.5	< .0001
WC (cm)	74.9 ± 9.9	75.3 ± 10.2	75.0 ± 10.0	93.0 ± 15.4	96.2 ± 17.8	94.2 ± 14.9	< .0001
Glucose (mg/dl)	91.5 ± 23.8	94.4 ± 28.0 **	92.6 ± 25.4	98.3 ± 22.9	100.5 ± 43.4	103.8 ± 40.2	< .0001
Triglycerides (mg/dl)	108.2 ± 66.6	112.3 ± 70.9	109.7 ± 68.2	143.6 ± 92.1	101.3 ± 59.4	150.6 ± 96.1	< .0001
HDL (mg/dl)	59.7 ± 14.2	58.5 ± 13.7 **	59.2 ± 14.0	59.0 ± 17.0	56.5 ± 17.4	54.4 ± 14.4	< .0001
LDL (mg/dl)	112.7 ± 27.1	115.6 ± 27.1 **	117.0 ± 26.7	121.6 ± 35.2	118.1 ± 37.4	112.7 ± 31.6	< .0001
Total Cholesterol (mg/dl)	182.8 ± 37.6	184.6 ± 36.6	182.5 ± 36.1	208.1 ± 41.7	194.5 ± 42.5	197.2 ± 41.7	< .0001
SBP (mm Hg)	110.3 ± 17.9	112.1 ± 17.5 **	110.9 ± 17.8	123.9 ± 22.7	123.3 ± 21.0	117.6 ± 20.6	< .0001
DBP (mm Hg)	71.7 ± 10.8	72.3 ± 10.5	71.9 ± 10.7	69.2 ± 11.4	71.0 ± 13.0	67.9 ± 10.8	< .0001
BMI (kg/m^2^)	22.5 ± 3.5	22.8 ± 4.0	22.6 ± 3.7	27.4 ± 6.4	30.0 ± 7.8	28.3 ± 6.3	< .0001
Weight (Kg)	55.7 ± 8.9	55.9 ± 9.8	55.8 ± 9.2	72.6 ± 17.4	80.1 ± 20.8	70.4 ± 17.0	< .0001
Height (cm)	157.3 ± 5.3	156.7 ± 5.2 **	157.1 ± 5.3	162.6 ± 6.8	163.2 ± 6.3	157.7 ± 6.2	< .0001
HT drug (%)	7.4	7.0	7.2	12.2	5.2	6.4	< .0001
DM drug (%)	2.7	3.0	2.8	5.0	8.7	9.5	< .0001
Lipid drug (%)	1.7	1.7	1.7	15.0	6.9	10.2	< .0001
Current smoker ^¥^(%)	3.0	2.2	2.7	19.7	21.7	15.5	< .0001

WC: waist circumference; HDL: high-density lipoprotein; LDL: low-density lipoprotein; SBP: systolic blood pressure; DBP: diastolic blood pressure;

BMI: body mass index.

^† ^At significant level α = 0.05, *p*-value < 0.05 was used for ANOVA for differences in means and chi-square test for proportions among four different ethnic groups; *p*-value < 0.05** was used for two-sample t-test were used for comparing means and proportions between urban and rural Taiwanese.

^‡ ^LDL is calculated from measured values of total cholesterol, triglycerides, and HDL according to the Friedewald calculation for US NHANES 2001-2002: [LDL] = [total cholesterol] - [HDL] - [triglycerides/5] where all values are expressed in mg/dL. The calculation is valid for triglycerides less than or equal to 400 mg/dL.

^¥ ^Current smokers were considered to subjects who have a life-time smoking experience of ≥ 100 cigarettes and currently smoke.

**Table 2:** Weighted prevalence of MetS and component risk factors by sex and ethnicity for Taiwan 2002TwHHH and US NHANES 2001-2002

	Ethnic Groups			
**Variables**	**Taiwanese**	**Non-Hispanic White**	**Non-Hispanic Black**	**Mexican- American**
**All**	**(N = 5786)**	**(N = 1077)**	**(N = 381)**	**(N = 461)**

Abdominal Obesity (NCEP)* ^¶^	29.8	48.5	46.9	43.1
Abdominal Obesity (IDF)^§ ¶^	29.8	71.1	62.6	67.8
High Blood Pressure**	29.8	39.7	45.8	21.7
Low HDL†	22.8	33.7	32.0	41.2
High Glucose‡	10.0	36.2	26.7	42.3
High Triglycerides^¥^	26.3	34.9	18.8	34.8
NCEP MetS	10.8	34.8	28.4	32.8
IDF MetS	8.6	39.9	31.5	35.2
**Males**	(N = 2827)	(N = 555)	(N = 196)	(N = 249)
Abdominal Obesity (NCEP)* ^¶^	29.2	39.9	29.3	34.0
Abdominal Obesity (IDF) ^§ ¶^	29.2	67.1	49.6	63.0
High Blood Pressure**	35.2	41.9	55.3	38.5
Low HDL†	21.1	32.9	21.7	34.6
High Glucose‡	10.5	46.1	31.3	60.6
High Triglycerides^¥^	33.2	39.0	23.5	43.0
NCEP MetS	11.2	36.7	26.6	39.5
IDF MetS	9.2	44.6	29.1	42.5
**Females**	(N = 2959)	(N = 522)	(N = 185)	(N = 212)
Abdominal Obesity (NCEP)* ^¶^	31.2	51.8	67.5	66.0
Abdominal Obesity (IDF) ^§ ¶^	31.2	72.2	83.2	86.1
High Blood Pressure**	25.0	36.1	50.3	32.4
Low HDL†	24.9	35.1	44.1	50.0
High Glucose‡	9.7	25.3	32.0	41.1
High Triglycerides^¥^	20.5	27.8	16.6	31.6
NCEP MetS	11.0	30.0	37.5	45.7
IDF MetS	8.3	33.2	41.1	47.0

Abbreviations are as footnoted in Table 1.

^¶ ^Males waist circumference ≥90 cm; females waist circumference ≥80 cm for Taiwanese population;

* NCEP: males waist circumference > 102 cm; female waist circumference > 88 cm;

^§ ^IDF: males waist circumference ≥94 cm; females waist circumference ≥80 cm

** Systolic ≥ 130 mmHg or diastolic ≥85 mmHg or on anti-hypertensive medication

† Males fasting HDL < 40 mg/dL; females fasting HDL < 50 mg/dL

‡ Fasting glucose ≥110 mg/dL or on glucose-lowering medication

^¥ ^Triglycerides ≥150 (mg/dl).

In regard to urbanization, the analysis was only possible for Taiwanese (Table 1). There were few biologically meaningful differences in the subject characteristics, although, large sample sizes allowed significance to be seen with some minor differences. In general, rural subjects had less satisfactory MetS related biological characteristics. Medication use for hypertension, diabetes and hyerlipidaemia did not differ between the 2 settings or by gender.

We have not shown the findings for physical activity or alcohol consumption because of the high frequencies (>20%) of missing values in NHANES. However, in Taiwan, this was not the case (missing values < 5%).

Pearson correlations are shown in Table 3. Although not a MetS component, we have included BMI as well as WC because of the interest in the intercorrelation between BMI and WC.

**Table 3:** Pearson Correlations of the MetS Components for Taiwanese, Non- Hispanic White, Non-Hispanic Blacks and Mexican-Americans

Variable	Ethnic Groups	WC	HDL	TG	FPG	SBP	DBP
BMI	Taiwanese	0.75*	-0.20*	0.41*	0.23*	0.36*	0.39*
	Rural	0.75*	-0.28*	0.47*	0.28*	0.46*	0.45*
	Urban	0.75*	-0.24*	0.45*	0.27*	0.41*	0.43*
	Non- Hispanic White	0.88*	-0.29*	0.30*	0.24*	0.14*	0.10^†^
	Non-Hispanic Blacks	0.91*	-0.21*	0.32*	0.24*	0.25*	0.11^‡^
	Mexican-Americans	0.90*	-0.23*	0.37*	0.20*	0.21*	0.19*

WC	Taiwanese		-0.25*	0.46*	0.27*	0.43*	0.44*
	Rural		-0.28*	0.47*	0.28*	0.46*	0.45*
	Urban		-0.24*	0.45*	0.27*	0.41*	0.43*
	Non- Hispanic White		-0.36*	0.40*	0.32*	0.20*	0.09^†^
	Non-Hispanic Blacks		-0.27*	0.41*	0.32*	0.32*	0.18^†^
	Mexican-Americans		-0.26*	0.43*	0.25*	0.30*	0.15^†^

HDL	Taiwanese			-0.41*	-0.08*	-0.11*	-0.12*
	Rural			-0.46*	-0.09*	-0.11*	-0.12*
	Urban			-0.38*	-0.07*	-0.09*	-0.12*
	Non- Hispanic White			-0.40*	-0.18*	-0.05	-0.10^†^
	Non-Hispanic Blacks			-0.32*	-0.18*	-0.01	-0.10^‡^
	Mexican-Americans			-0.36*	-0.24*	-0.06	-0.07

TG	Taiwanese				0.28*	0.33*	0.34*
	Rural				0.26*	0.32*	0.32*
	Urban				0.28*	0.34*	0.35*
	Non- Hispanic White				0.26*	0.15*	0.07^‡^
	Non-Hispanic Blacks				0.35*	0.22*	0.14^‡^
	Mexican-Americans				0.27*	0.23*	0.18*

FPG	Taiwanese					0.28*	0.22*
	Rural					0.29*	0.23*
	Urban					0.27*	0.21*
	Non- Hispanic White					0.24*	0.02
	Non-Hispanic Blacks					0.32*	0.15^‡^
	Mexican-Americans					0.33*	0.23*

SBP	Taiwanese						0.76*
	Rural						0.76*
	Urban						0.77*
	Non- Hispanic White						0.29*
	Non-Hispanic Blacks						0.54*
	Mexican-Americans						0.42*

All variables are log-transformed

Subjects with missing values of WC, HDL, TG, FPG, SBP or DBP < 30 mmHg and age less than 18 yrs old are deleted

SBP: systolic blood pressure; DBP: diastolic blood pressure

*P < 0.001

^†^*P *< 0.01

^‡^*P *< 0.05

The correlation between BMI and WC is less in Taiwanese (r = 0.75) than in US ethnic groups (r = 0.88, 0.91 and 0.90 for Non-Hispanic White: NHW; Non-Hispanic Blacks: NHB and Mexican American: MA, respectively). With the exception of blood pressure, the remaining correlations between the metabolic syndrome components are similar for the various ethnic groups.

### Exploratory factor analysis

**Figure 1:**
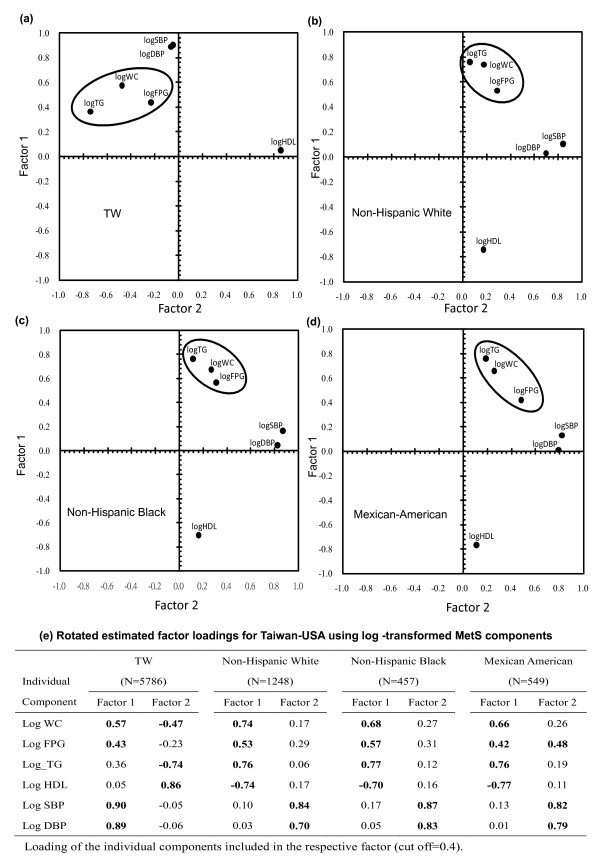
**Factor loading pairs of the MetS components from PCA after rotation for different ethnic groups**.

For both the TW3HHH and NHANES datasets, all the distributions of the six components were skewed to the left. Therefore, a log-transformation was applied to each of the six components before analysis, so that the component variables were approximately normally distributed. Figure 1a-1d presents the score plots from PCA after rotation of the ethnic groups separately. The data points with similar factor structures were closely located as a cluster whereas those with divergent patterns were located further apart. They were classified according to the position of the corresponding coordinates with respect to the factor axis after rotation. As shown in Figure 1a-1d, for all the four ethnicity groups, the score plot shows that the data points were clustered into three groups: IER (Impaired Energy Regulation) components (WC, FPG, TG), systolic and diastolic blood pressures (SBP, DBP), and HDL-cholesterol. For the three US ethnic subgroups, factor 1 was mainly determined by the IER components and low HDL-cholesterol, and factor 2 was related to the BPs components; whereas for Taiwanese the IER components were determinant for both factors 1 and 2, and hypertension and HDL-cholesterol were related to factors 1 and 2 respectively (Figure 1e).

In the case of Taiwan, the IER cluster was closer to the BP cluster than in the US ethnic groups.

**Figure 2:**
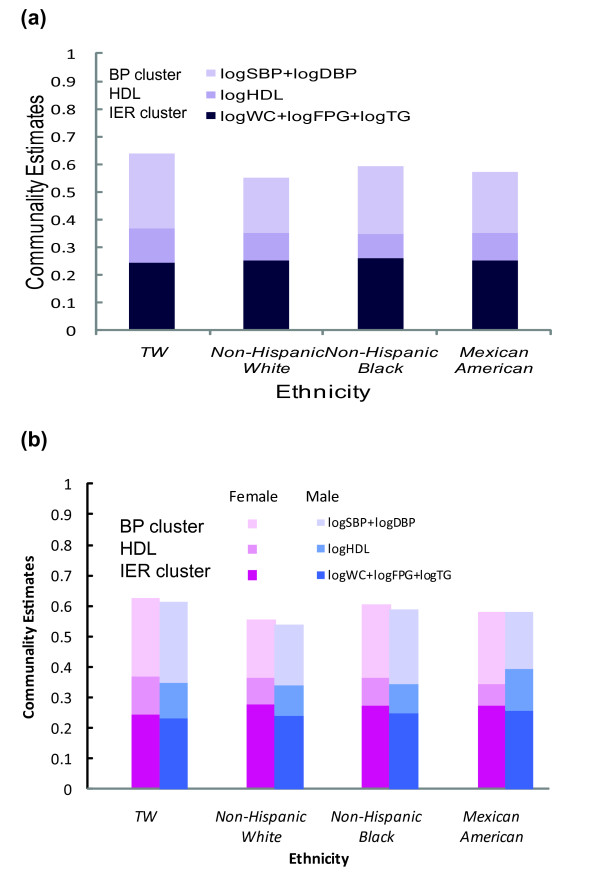
**Communality estimates of factor analysis across four ethnic groups: (a) overall; (b) by gender**.

Figure 2 illustrates the total variance explained by the first two factors and relative variance compositions of the IER components, BPs, and HDL-cholesterol across the four ethnic groups, with specific percentages given in Table 4. The first two factors accounted for 55.2% (Caucasian) to 63.7% (Taiwanese) of the total variances. As shown in figure 2, the proportions of variance explained by the IER components were relatively homogeneous for different ethnic subgroups, with absolute percentages ranging from 24.6% (Taiwanese) to 25.9% (non-Hispanic black) and relative percentages ranging from 38.5% to 45.8%. However, the proportions of variance explained by BPs and HDL-cholesterol were somewhat heterogeneous. For example, the absolute percentages of hypertension ranged between 20.1% (non-Hispanic white) and 26.8% (Taiwanese). The invariance pattern of the IER components was similar across the four ethnic subgroups when stratified by gender. However, males had slightly lower proportions of the IER components than females for all the four ethnic groups.

**Table 4:** Proportions of variances explained by the IER and other components of the first two factors from PCA across the four ethnic groups: overall and by gender*

Overall								
**Communality** **(%)**	**Taiwanese** **(N = 5786)**		**Non-Hispanic White** **(N = 1248)**		**Non-Hispanic Black** **(N = 457)**		**Mexican-American** **(N = 549)**	
	**Absolute**	**Relative**	**Absolute**	**Relative**	**Absolute**	**Relative**	**Absolute**	**Relative**

logSBP+logDBP	26.8 (26.1, 27.3)	42.0 (41.0, 43.0)	20.1 (18.5, 21.7)	36.5 (33.7, 38.9)	24.5 (22.5, 26.0)	41.2 (38.1, 44.2)	22.0 (20.1, 23.6)	38.4 (35.2, 41.0)
logHDL	12.4 (11.7, 12.9)	19.5 (18.5, 20.2)	9.7 (8.6, 10.8)	17.7 (15.5, 19.5)	8.7 (6.2, 10.8)	14.7 (10.7, 18.0)	10.0 (8.1, 11.7)	17.4 (14.2, 20.2)
logWC+logFPG+logTG	24.6 (24.0, 25.2)	38.5 (37.9, 39.2)	25.3 (24.3, 26.7)	45.8 (44.2, 47.8)	25.9 (24.5, 28.1)	43.9 (41.7, 46.7)	25.3 (23.5, 27.9)	44.2 (41.6, 48.1)
Factor1+Factor2	63.7 (63.0, 64.5)	100	55.2 (53.9, 57.0)	100	59.1 (57.0, 61.8)	100	57.5 (55.2, 59.8)	100

**Male**								

**Communality** **(%)**	**Taiwanese** **(N = 2827)**		**Non-HispanicWhite** **(N = 602)**		**Non-Hispanic Black** **(N = 226)**		**Mexican-American** **(N = 266)**	
	**Absolute**	**Relative**	**Absolute**	**Relative**	**Absolute**	**Relative**	**Absolute**	**Relative**

logSBP+logDBP	26.6 (25.9, 27.3)	43.4 (42.2, 44.5)	20.1 (17.5, 21.8)	37.3 (32.5, 39.8)	24.2 (20.3,26.7)	41.3 (34.6,45.0)	18.8 (15.8, 21.4)	32.8 (27.7, 36.3)
logHDL	11.5 (10.8, 12.1)	18.7 (17.6, 19.6)	9.8 (7.9, 12.2)	18.1 (14.6, 22.6)	9.5 (5.5, 12.8)	16.1 (9.4, 21.3)	13.4 (11.7, 14.2)	23.1 (19.9, 24.6)
logWC+logFPG+logTG	23.3 (22.4, 24.3)	37.9 (37.0, 39.0)	24.0 (22.2, 27.2)	44.5 (41.6, 49.3)	25.0 (23.3,28.6)	42.6 (40.0,47.7)	25.8 (23.7, 28.9)	44.1 (41.8, 48.9)
Factor1+Factor2	61.3 (60.4, 62.4)	100	53.9 (52.3, 56.7)	100	58.7 (55.6,62.9)	100	58.0 (55.1, 61.5)	100

**Female**								

**Communality** **(%)**	**Taiwanese** **(N = 2959)**		**Non-HispanicWhite** **(N = 646)**		**Non-Hispanic Black** **(N = 231)**		**Mexican-American** **(N = 283)**	
	**Absolute**	**Relative**	**Absolute**	**Relative**	**Absolute**	**Relative**	**Absolute**	**Relative**

logSBP+logDBP	25.7 (24.9, 26.5)	41.2 (39.7, 42.5)	19.1 (16.9, 21.5)	34.5 (30.4, 37.8)	24.2 (22.2, 26.2)	39.8 (36.0, 43.7)	23.7 (21.7, 25.3)	40.6 (37.4, 43.5)
logHDL	12.4 (11.2, 13.4)	19.8 (17.9, 21.4)	8.5 (7.0, 10.0)	15.4 (12.5, 17.8)	9.0 (5.5, 11.6)	14.8 (9.4, 18.4)	7.2 (4.2, 9.8)	12.3 (7.4, 16.6)
logWC+logFPG+logTG	24.4 (23.3, 25.6)	39.1 (37.8, 40.4)	27.9 (26.5, 30.2)	50.2 (47.6, 53.3)	27.5 (25.6, 30.3)	45.4 (42.9, 49.0)	27.4 (24.5, 31.1)	47.1 (42.6, 52.9)
Factor1+Factor2	62.5 (61.4, 63.7)	100	55.6 (54.2, 58.3)	100	60.7 (57.8, 64.7)	100	58.2 (55.6, 61.6)	100

* The numbers in parentheses are the 95% CIs obtained from 1000 bootstrap resamplings

**Figure 3:**
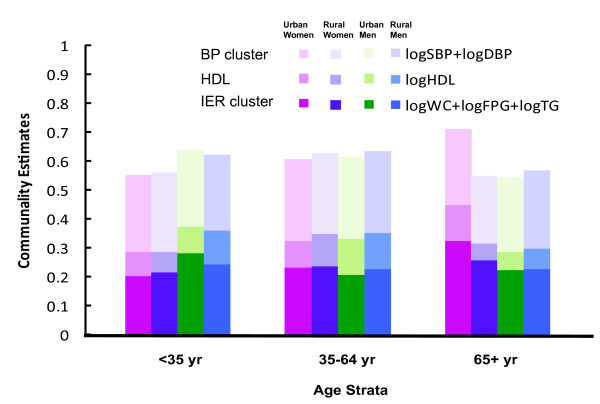
**Communality estimates of factor analysis across urban and rural Taiwanese by gender and age strata**.

In contrast, females tended to have lower communalities in BPs than males except for Mexican-Americans, for whom the females had substantially higher communality. The 95% CIs obtained from bootstrap resamplings were reasonably narrow and homogeneous for all the clustered components across the four ethnic groups as well as the males and females separately, with Taiwanese having the narrowest bounds possibly due to the relatively large sample size (Figure 3, Table 4).

Table 5 shows the subject comparisons for Taiwanese by age strata (< 35, 35-65, and. ≥ 65), by gender and by urban-rural locality. There are generally unfavourable differences for younger and middle-aged rural compared with urban subject, but not for elderly people. Medication usage is greater for the elderly, whether urban or rural, as expected. Smoking is markedly less for women than men in all age groups and in both urban and rural settings. When considered by gender, there are no differences between urban and rural dwellers for smoking.

**Table 5:** Age-specific comparisons of the MetS components of Taiwanese with different urbanization status

	< 35 yr		35-64 yr		65 + yr	
	
Variables	Urban	Rural	Urban	Rural	Urban	Rural
		
All	N = 1246	N = 769	N = 2056	N = 1112	N = 348	N = 255
Age (years)	26.0 ± 5.2	26.2 ± 5.1	47.1 ± 7.8	47.6 ± 8.3	72.3 ± 5.3	72.7 ± 5.7
WC (cm)	75.3 ± 11.0	75.4 ± 11.0	80.8 ± 10.8	81.9 ± 10.6 *	85.5 ± 9.9	85.0 ± 9.5
Glucose (mg/dl)	85.3 ± 13.4	87.0 ± 12.5 *	95.1 ± 28.5	99.3 ± 33.9 *	102.5 ± 33.7	104.6 ± 35.5
Triglycerides(mg/dl)	99.9 ± 59.5	111.2 ± 70.7 *	133.7 ± 86.8	142.6 ±100.3 *	142.4 ± 77.2	149.1 ± 92.3
HDL (mg/dl)	54.7 ± 12.6	53.4 ± 13.3	56.9 ± 15.8	55.4 ± 15.4 *	56.1 ± 15.8	54.0 ± 15.9
LDL (mg/dl)	102.6 ± 23.4	107.8 ± 24.5 *	120.0 ± 26.3	120.7 ± 26.6	126.3 ± 27.4	123.4 ± 28.3
Total Cholesterol(mg/dl)	166.3 ± 31.5	171.2 ± 32.6 *	190.3 ± 36.3	190.1 ± 36.1	196.5 ± 36.9	191.4 ± 39.5
SBP (mm Hg)	105.7 ± 11.8	107.8 ± 12.3 *	115.3 ± 16.9	118.6 ± 17.2 *	133.2 ± 18.7	133.1 ± 18.3
DBP (mm Hg)	70.0 ± 9.4	71.0 ± 9.6 *	76.7 ± 11.4	77.6 ± 11.5 *	78.1 ± 10.8	78.3 ± 10.9
BMI (kg/m^2^)	21.9 ± 3.6	22.3 ± 3.8 *	23.7 ± 3.3	24.0 ± 3.6 *	23.8 ± 3.2	23.4 ± 3.1
Weight (Kg)	60.3 ± 12.5	60.4 ± 12.8	62.2 ± 11.1	62.8 ± 11.3	61.6 ± 10.0	60.5 ± 9.7
Height (cm)	165.4 ± 8.1	164.2 ± 8.0 *	161.7 ± 7.7	161.4 ± 7.6	160.8 ± 8.4	160.7 ± 7.9
HT drug (%)	0.2	0	7.6	7.8	32.8	33.7
DM drug (%)	0.2	0	3.4	3.8	12.1	11.4
Lipid drug (%)	0.08	0.13	2.0	1.5	5.7	6.7
Current smokers ^¥ ^(%)	16.9	17.8	20.2	24.1 *	17.8	25.9 *

**Males**	**N = 611**	**N = 360**	**N = 942**	**N = 559**	**N = 193**	**N = 162**
Age (years)	25.7 ± 5.2	25.9 ± 5.2	47.2 ± 7.8	47.7 ± 8.5	72.6 ± 5.1	72.7 ± 5.4
WC (cm)	80.5 ± 10.9	80.7 ± 10.9	86.1 ± 9.9	86.2 ± 9.6	86.9 ± 10.2	86.6 ± 8.8
Glucose (mg/dl)	86.8 ± 15.5	87.5 ± 14.6	96.6 ± 32.3	100.2 ± 34.0	99.5 ± 27.4	103.8 ± 35.8
Triglycerides(mg/dl)	114.1 ± 68.0	132.5 ± 85.6 *	156.1 ± 98.9	164.4 ± 114.7 *	135.6 ± 72.0	148.4 ± 92.5
HDL (mg/dl)	51.5 ± 12.3	49.5 ± 13.0 *	52.2 ± 15.4	51.2 ± 15.5	53.4 ± 15.4	51.0 ± 15.6
LDL (mg/dl)	106.1 ± 25.5	109.3 ± 25.1	122.4 ± 25.4	121.2 ± 26.2	123.4 ± 26.3	121.2 ± 27.5
Total Cholesterol (mg/dl)	168.1 ± 34.4	170.8 ± 33.0	190.5 ± 34.2	188.7 ± 36.1	189.9 ± 34.1	186.0 ± 38.7
SBP (mm Hg)	111.5 ± 11.1	114.1 ± 11.3 *	118.4 ± 16.3	121.2 ± 16.7 *	132.2 ± 16.6	133.3 ± 18.9
DBP (mm Hg)	73.9 ± 8.9	74.9 ± 9.4	80.0 ± 11.1	80.2 ± 11.5	78.2 ± 10.6	79.1 ± 11.4
BMI (kg/m^2^)	22.9 ± 3.7	23.4 ± 3.9 *	24.3 ± 3.2	24.4 ± 3.1	23.3 ± 2.9	23.4 ± 2.8
Weight (Kg)	67.5 ± 11.9	68.1 ± 12.0	68.5 ± 10.1	67.9 ± 10.2	64.6 ± 9.9	63.7 ± 8.3
Height (cm)	171.5 ± 5.9	170.5 ± 5.7 *	167.7 ± 5.7	166.8 ± 5.7 *	166.3 ± 5.9	165.0 ± 5.3 *
HT drug (%)	0.3	0	7.0	7.2	33.7	36.4
DM drug (%)	0.3	0	3.6	4.3	13.5	9.3
Lipid drug (%)	0.2	0.3	2.0	1.3	5.2	5.6
Current smokers ^¥ ^(%)	31.4	35.8	40.3	46.0	30.6	38.3

**Females**	**N = 635**	**N = 409**	**N = 1114**	**N = 553**	**N = 155**	**N = 93**
Age (years)	26.2 ± 5.2	26.4 ± 5.1	47.1 ± 7.9	47.5 ± 8.1	71.8 ± 5.6	72.8 ± 6.2
WC (cm)	70.3 ± 8.7	70.7 ± 8.8	76.3 ± 9.4	77.5 ± 9.8 *	83.6 ± 9.2	82.2 ± 10.1
Glucose (mg/dl)	83.9 ± 10.7	86.5 ± 10.3 *	93.8 ± 24.7	98.4 ± 33.7 *	106.2 ± 40.0	105.8 ± 35.0
Triglycerides (mg/dl)	86.3 ± 46.0	92.5 ± 47.0 *	114.8 ± 69.8	120.4 ± 77.1	150.8 ± 82.7	150.4 ± 92.3
HDL (mg/dl)	57.7 ± 12.1	56.8 ± 12.7 *	60.9 ± 14.9	59.6 ± 14.1	59.4 ± 15.7	59.3 ± 15.0
LDL (mg/dl)	99.3 ± 20.7	106.5 ± 24.0	118.0 ± 26.9	120.3 ± 27.0	129.9 ± 28.5	127.2 ± 29.3
Total Cholesterol (mg/dl)	164.5 ± 28.3	171.5 ± 32.3 *	190.2 ± 37.9	191.6 ± 36.1	204.6 ± 38.8	200.9 ± 39.4
SBP (mm Hg)	100.1 ± 9.6	102.2 ± 10.3 *	112.7 ± 17.1	115.9 ± 17.3 *	134.4 ± 21.0	132.7 ± 17.5
DBP (mm Hg)	66.3 ± 8.2	67.6 ± 8.4 *	73.8 ± 10.8	75.0 ± 10.8 *	77.8 ± 11.0	76.8 ± 9.7
BMI (kg/m^2^)	21.0 ± 3.2	21.3 ± 3.6	23.2 ± 3.4	23.7 ± 4.0 *	24.4 ± 3.5	23.5 ± 3.7 *
Weight (Kg)	53.4 ± 8.6	53.6 ± 9.2	56.8 ± 8.7	57.6 ± 9.9	57.8 ± 8.6	55.0 ± 9.5 *
Height (cm)	159.5 ± 5.0	158.5 ± 4.9 *	156.6 ± 5.0	155.9 ± 4.8 *	154.0 ± 5.6	153.0 ± 5.6
HT drug (%)	0	0	8.2	8.5	31.6	29.0
DM drug (%)	0	0	3.2	3.3	10.3	15.1
Lipid drug (%)	0	0	2.0	1.8	6.5	8.6
Current smokers ^¥ ^(%)	3.0	2.0	3.1	2.0	1.9	4.3

^† ^At significant level α = 0.05, *p*-value < 0.05 (*) was used for two-sample *t-*test were used for comparing means between urban and rural Taiwanese in each gender and age-specific strata.

^¥ ^Current smokers were considered to subjects who have a life-time smoking experience of ≥ 100 cigarettes and currently smoke

In Figure 3 we show the absolute cumulative variances (Communality Estimates) for MetS component clusters obtained from factor analysis where the cut-points for factor loadings were 0.40. These correspond to the age, gender and locality settings represented in Table 5. Two MetS clusters (WC, FPG, TG and SBP with DBP) and HDL together explain about 50-60% of the communality estimates for the MetS, and the IER cluster explains about 25%, as these clusters do when the analyses are by ethnicity as shown in Table 4 and in figures 1 and 2. The exception is that of urban women where 3 factors emerge (data not shown) and the IER cluster does so more strongly (32.5% of the communality estimates compared with 25.8% for the rural women); in the case of urban women, the same 2 collective clusters and HDL explain more than 70% of the communality estimates for the MetS. These findings indicate that an IER core of the MetS components exists irrespective of ethnicity, gender, age or locality, although more pronounced for older urban women.

The factor analysis for elderly Taiwanese (≥ 65 y) is similar to a factor analysis of the MetS in elderly Swedes (≥ 75 y) [29] where a core of WC, FPG, TG and Inverse HDL constituted a Metabolic Factor and, with a BP Factor, accounted for most of the variance (data not shown). Although the communality estimates are not available to us for the Swedish elderly who were located in a provincial setting, we can say that elderly men in Sweden and Taiwan have comparable factor loadings for the various MetS components [29] Rural Taiwanese women, but not Urban Taiwanese women, had a Metabolic factor and a BP factor like provincial Swedish women.

## Discussion

### Consistent component core

This study utilized factor analysis to discover a consistent component core as a likely core of the metabolic syndrome. We found a similar pattern in different ethnic groups. It confirms our hypothesis about IER. Wijndaele et al. [30] also used factor analysis to construct scores for the metabolic syndrome. They obtained two factors based on the components of the metabolic syndrome. They then multiplied each component by factor loading and weighed the sum by the relative contribution in the proportion of variance explained by each factor. They called it the "continuous metabolic syndrome risk score". Later on, they used this score as a dependent variable and investigated the effects of sedentary behaviors and leisure time physical activity on it [31]. They did not investigate the clustering nature of each component in detail. Instead, they used the score directly. We also obtained two factors for different ethnic groups. With an in depth examination of the clustering nature of each component, we found that there was a component aggregate in the metabolic syndrome, which we have referred to as IER, a core to the conventional MetS.

Öhrvik et al. [29] have explored factor analysis of the MetS in an elderly Swedish population. They used the reciprocal of HDL and log transformation of FPG and TG and found two factors. A metabolic factor with our three IER components and a BP factor in men and women. We have analyzed the elderly Taiwanese data in the same way and find similar results for men, and for rural women, but not for urban women, who seem to have enhanced the 'IER' core seen in the younger population in its various settings and have 3 rather than 2 factors (Figure 3) [29]. We find this age, gender and urban-rural differential of interest since it may indicate a biological survival strategy or, conversely, be a signal of potential demise, or just relate to ageing in some way, depending on what comes first and its predictive power for future health. This is a question we expect to pursue in future data-linkage studies on morbidity and mortality outcomes.

At present we can say that ethnicity, and urbanisation and, with the exception of elderly women in urban settings, display a common core to the MetS which could reflect IER. Where this does not apply may provide clues to the pathogenesis and consequences of the MetS. An example of this could be the report of Malan et al (2008) [18] that the expression of the MetS syndrome by setting (urban-rural) may depend on an individual's coping skills.

### Unifying capacity of IER

Various attempts have been made to provide a mechanistic construct on the many observations of the MetS cluster of 6 components with insulin resistance being the earliest and most preferred. For example, Alberti and colleagues recommended one single set of cut points for all components except waist circumference [32]. Nevertheless, it has begged the question as to what is fundamentally going on in people who manifest it, especially in regard to its genotypic, phenotypic or, more recently, its epigenetic basis. By the conceptual separation of IER from the rest of the MetS, it is possible to consider its inputs and consequences in more systematic ways. We would advance the view, supported by the present enquiry into the phenomena in the US and Taiwan, that energy dysnutrition with its environmental inputs of energy intake and expenditure and its genomic determinants are at the core of the MetS. Then, depending on the disorder or disease which might be consequent upon it, the ways of rounding out the syndrome, now referred to as IER, might be several. Most usually, it is the addition of other cardiovascular risk factors, like BP and hyperLDL/HDLemia which have strengthened the cluster in regard to its prediction of macrovascular disease [26,29]; or of insulin or its sensitivity when it comes to pre-diabetes or diabetes. Increasingly, there is interest in other connections that the MetS has with other metabolic disorders like hyperuricaemia [24,33], inflammatory disorders [34], hematological disorders [35] and neoplastic disorders [8]. As a matter of fact, these various linkages may also have IER at their basis.

A somewhat similar conceptualization of the patho-physiological basis of the MetS has been advanced by others [36,37]. Despres and Lemieux considered that fat maldistribution or 'dysfunctional adipose tissue' is of central importance in the diagnosis of MetS [36]. What we have done is, through factor analysis, found that three components of the MetS underpin this way of thinking. Moreover, we perceive this has been, at a more fundamental level, a measure of energy dysnutrition with its associated anatomical logic in the splanchnic region.

If additional components, like insulin, are included in the MetS definition, they will direct the unifying concept accordingly, in this case towards insulin resistance [38]. In this way, what might constitute the basis of the insulin resistance, such as IER, is obscured. In our analysis, we have been able to discern a phenomenon located in less than 6, and not more than 6, MetS components. Based on a Caucasian twin study of metabolic risk factors (MRF) for diabetes and cardiovascular disease, Peeters et al. argued that a focus on MRF specific loci may be a more useful approach to the understanding of gene-environment risk than the MetS as a whole [39]. We agree that few MRFs may be more instructive than many and consider that those components which are additional to IER belong to various second order MRF phenomena.

While we have not formally assessed insulin resistance, fasting glucose represents a partial measure of it, at least insofar as nocturnal gluconeogenesis and its determination by FFA flux in the splanchnic circulation is concerned. Seen this way, insulin resistance may be embraced by the broader and unifying phenomenon of IER.

### EFA or CFA for Reconceptualisation of the Metabolic Syndrome?

We propose that IER components (WC, FPG, & TG) rather than the usual six components of MetS are the core metabolic disorder in the MetS, and have employed an exploratory factor analysis (EFA) to differentiate the clustering and inter-relationships among the 6 components of MetS. As the EFA has showed, this hypothesis is "confirmed" by the invariant (or homogeneous) pattern across four different ethnic groups. It might be asked why we did not employ a confirmatory factor analysis (CFA) to confirm our hypothesis at the beginning? It is the starting point for those authors who adopt the CFA approach [38,40,41] in attempting to identify a latent structure or factor(s) for the MetS. Our rationale was that first, since the 3 IER components were already observed to cluster from our datasets, we could explore them together with the other 3 components directly and let the data speak for themselves. Second, for those who adopted the CFA approach, the MetS is usually accepted as the underlying framework in their analysis; we did not want to do that, but rather question the validity of the present MetS to represent an integrated pathophysiological phenomenon. Ours is an attempt to break out of the circular thinking which has limited MetS research, in our view, and to search for a novel phenotype. Third, our paper attempts to re-conceptualize the MetS. It was therefore inappropriate to *manipulate *the datasets in accordance with a hypothesis and then test for it. Hence, we used an exploratory rather than a confirmatory approach.

As indicated, the conceptual IER segregates from the rest of the components of the MetS and provides a unifying capacity with which to consider inputs and consequences in more systematic ways. Greater sophistication in our hypothesis would involve reference to insinuated, but unobserved measurements (such as free fatty acid) or latent or partially captured factors (such as insulin resistance), at which point the CFA approach would be appropriate. Our findings provide a near-fundamental basis with which to take IER further through more structured hypotheses about phenotype, cellular biology and genomics. Such an enquiry would have public health and clinical relevance.

### Relation of the IER to other MetS components

The extent to which the non-IER MetS components and their health outcome significance differ between the ethnic groups studied is of interest We have noted in the results that, for Taiwan, the BP cluster is closer to the IER cluster than in the US ethnic populations. Furthermore, in the case of diastolic BP, where Taiwan has the highest values and prevalence, it is against a background of preference for a high Na/K molar ratio diet, and relatively high incidence of hemorrhagic stroke as featured in the annual Public Health reports. On the other hand, relatively greater North American lipoprotein cholesterol abnormalities (Tables 1 and 2) appear to accentuate the risk for atherosclerotic macrovascular disease amongst the 3 relevant ethnic groups in this study, as judged from other evidence. On the other hand, in at least some populations, the ability of the MetS to predict clinical outcomes of diabetes and cardiovascular disease is widely divergent, as in elderly Europeans where the prediction of the former greater exceeded that of the latter [42]. This would lend support to the notion that the impact of the IER MetS components might or might not depend on the non-IER MetS components and other factors.

A more analytical study of the representative Taiwanese subjects by age, gender and locality provides further insights into the core and second order of the MetS components (Table 5 and figure 3). With regard to age, most components (WC, glucose, triglycerides, SBP and DBP), except HDL cholesterol, are more compromised in older individuals, whether men or women, or whether urban or rural. However, the core continues to behave in a coherent way (Figure 3), except in older urban women where 3 factors emerge.

### Pathophysiology

Our segregation of the MetS into an energy-dependent core (IER) and other phenomena is consistent with emerging evidence on the regulation of cellular fuel metabolism and about mitochondrial function and disorder. In particular, the AMP-activated protein kinase cascade has been identified as a unifying system for energy control by Carling and others [43]. It also is involved in insulin signaling [44] and senses glycogen stores [45]. Another possibility which has been proposed by Bhopal and Rafnssson is that mitochondrial efficiency might explain susceptibility to adiposity and the metabolic syndrome, which is to say that ATP and heat generation might be involved, perhaps through mitochondrial DNA mutations characteristic of certain populations [46]. Since, in absolute values, our IER components are lower in Taiwanese than in any of the US populations, this is a difference which could represent mitochondrial efficiency differences. Aside from the likely pivotal role of exercise and energy intake in the modulation of IER as for the MetS itself [46], it may offer new approaches in the treatment of metabolic disorders [47]. Linked to this will be the integrity of mitochondrial energy metabolism and function [48,49].

### Health status of subjects

In both the US and Taiwan, the study populations were community-based and representative, not selected for health status and not institutionalised in this cross-sectional study. Their health characteristics are shown in part, by reported behaviours (eg tobacco smoking) and various risk factors, by the medication history, by anthropometry to detect body fatness and its distribution and by virtue of the components of the MetS itself. Although physical activity and alcohol intake were recorded, the frequency of missing values in the US ethnic groups precluded their reliable evaluation.

The interpretation of the medication use is difficult. First, it is some confirmation of a risk or disease state eg hypertension, hyperlipidaemia or diabetes. Second, it means the observed MetS components will have been somewhat corrected depending on compliance, affordability, access to health care and public health education. In these respects the US and Taiwan are strikingly different, since Taiwan has a universal health insurance scheme (which includes pharmaceuticals), with 99% of the population covered, and the US does not. Nevertheless, in both places, remoteness, culture, education, socio-economic circumstances and other equity issues are likely to have influenced MetS component profiles. For example, our data showed age and gender might have effects on MetS, especially on urban older women. Notwithstanding these considerations, the MetS core is robust by setting, health status and where other differences will be inevitable.

### Public health and clinical implications

If the MetS can be understood in clearer etiologic and pathogenetic terms, it will pave the way for more rational and supportable public health preventive strategies and clinical interventions.

It remains unclear as to how important the MetS is in the prediction of morbidity and mortality in different clinical settings and the understanding of IER may clarify this situation. From the public health point of view, the "physical activity transition" and its role in the genesis of MRFs as proposed by Katzmarzyk and Mason may operate principally through the IER pathway [46].

## Conclusions

Energy dysnutrition is a fundamental problem at all stages of economic development. It may operate at different levels from the environmental-human biology interface to every human cell, in turn to determine the likelihood of health and well-being. However, in public health and clinical practice, its measurement is available, not only by way of intake and expenditure with difficulty, but also by way of waist (or abdominal) circumference for abdominal fat stores and by fasting plasma glucose and triglycerides as fuels generated in the splanchnic circulation and region. These 3 components (IER) of the MetS cluster consistently across several ethnic groups in different localities; nevertheless, the phenomenon is not as pronounced in Taiwan as the US and its persistence into later life is less evident in urban Taiwanese women The IER cluster allows a step-wise first (core) and then second order approach to the understanding and application of the present form of the MetS, in relation to cardiovascular disease. This also allows for other variants of the MetS, more relevant, for example, to inflammatory or neoplastic disease, to be developed as second order phenomena. We are encouraged to take advantage of these findings in cohort and intervention studies, especially those which include genetic markers for energy metabolism, fat distribution, and various clinical outcomes.

## Abbreviations

AHA: American Heart Association criteria for metabolic syndrome in 2004; BMI: Body Mass Indices; BPs: systolic and diastolic blood pressures; CFA: confirmatory factor analysis; CI: confidence interval; DBP: diastolic blood pressure; EFA: Exploratory factor analysis; FFA: free fatty acids; FPG: fasting plasma glucose; HDL: high-density lipoprotein; IDF: International Diabetes Federation criteria for metabolic syndrome; IER: Impaired Energy Regulation; LDL: low-density lipoprotein; MRF: metabolic risk factors; MetS: metabolic syndrome; NCEP-ATP III: National Cholesterol Education Program-Adult Treatment Panel III criteria for metabolic syndrome; NHANES: National Health and Nutrition E1xamination Survey; NHIS: National Health Interview Survey; NHLB: National Heart Lung and Blood Institute; PC: principal components; PCA: PC analysis; PPS: probability proportional to their size; SBP: systolic blood pressure; TG: fasting triglycerides; TwSHHH: Taiwan Three High Prevalence Survey on Hypertension, Hyperglycemia, and Hyperlipidemia; VLDL: very low density lipoprotein; WC: waist circumference.

## Competing interests

The authors declare that they have no competing interests.

## Authors' contributions

MLW, HYC, CCC and CCH were involved in the conception and design. WCC WSW and CCC were involved in the analysis and interpretation of the data. MLW, HYC, CCC and CCH drafted the article. MLW, HYC and CAH were responsible for critical revision of the article for intellectual content. MLW, HYC, CCC, CCH, WCC, WSW and CAH gave final approval of the article. HYC, CCC WCC, and WSW provided statistical expertise. HYC, WCC and WSW were involved in the collection, where relevant, and assembly of the data. All authors read and approved the final manuscript.

## Pre-publication history

The pre-publication history for this paper can be accessed here:

http://www.biomedcentral.com/1472-6823/10/11/prepub
